# Recurrent Fever and Biliary Ectasia Leading to the Diagnosis of Caroli Syndrome: A Diagnostic Challenge

**DOI:** 10.7759/cureus.111646

**Published:** 2026-06-28

**Authors:** Muhammad Usman, Abdul Basit, Bilal Ahmad Buttar, Muhammad Aftab Ahmad, Laiba Murtaza

**Affiliations:** 1 Internal Medicine, Mercy Hospital Fort Smith, Fort Smith, USA; 2 Internal Medicine, Arkansas College of Osteopathic Medicine, Fort Smith, USA; 3 Internal Medicine, King Edward Medical University, Lahore, PAK

**Keywords:** caroli disease, caroli syndrome, congenital hepatic fibrosis, fever of unknown origin, hepatobiliary disorders

## Abstract

Caroli syndrome (CS) is a rare congenital fibropolycystic liver disorder characterized by non-obstructive intrahepatic bile duct dilatation and congenital hepatic fibrosis, often masquerading as fever of unknown origin. A 38-year-old Hispanic male presented with four weeks of intermittent high-grade fevers and right upper quadrant pain following recent travel to Mexico. An extensive infectious workup was unrevealing, while inflammatory markers were markedly elevated despite normal liver enzyme levels. Cross-sectional imaging revealed multiple intrahepatic cystic dilatations with biliary ectasia, and liver biopsy confirmed congenital hepatic fibrosis, establishing the diagnosis of CS. He defervesced rapidly on empirical broad-spectrum antibiotics. CS should be considered in patients with recurrent unexplained fevers and biliary abnormalities, even when liver enzyme levels are normal, to enable timely diagnosis and surveillance for long-term complications.

## Introduction

Caroli syndrome (CS) is a rare congenital disorder of the biliary tree, classically defined by non-obstructive saccular dilatation of the intrahepatic bile ducts with congenital hepatic fibrosis [1]. It arises from ductal plate malformation during embryogenesis and is a ciliopathy linked to polycystic kidney and hepatic disease 1 (PKHD1) mutations [2]. With an estimated incidence of one per million, it remains a relatively uncommon entity [1,2].

What makes CS particularly challenging is its subtle and insidious presentation. Many patients reach early adulthood before symptoms emerge, and even then, they are often nonspecific: recurrent fevers, right upper quadrant (RUQ) discomfort, or episodes mistaken for unrelated infections [3]. Biochemical markers often remain deceptively normal despite structural disease, leading to delays in diagnosis and recurrent hospitalizations [3,4].

Early recognition is vital to manage chronic stasis, portal hypertension, and the increased risk of cholangiocarcinoma, with reported malignancy rates ranging from 6.6% to 37% [5]. Imaging suggests the diagnosis, but histological confirmation of congenital hepatic fibrosis distinguishes CS from Caroli disease [4,5]. Here, we present a 38-year-old Hispanic male with recurrent hospitalizations for high-grade fevers and RUQ pain who was ultimately diagnosed with CS.

## Case presentation

A 38-year-old Hispanic male with an unremarkable past medical history presented to the emergency department with intermittent episodes of high-grade fever (103-104 °F) for the past four weeks. The patient reported that he had been taking acetaminophen with symptomatic relief. Associated symptoms included diaphoresis, headache, and nausea without vomiting. A few days before presentation, he also developed intermittent RUQ abdominal pain. He reported that approximately 2.5 months earlier, he had visited relatives in Mexico, where he stayed for about one month. He denied any tick bites, mosquito bites, or sick contacts. He reported consuming undercooked pork and getting a new tattoo on his right upper extremity during his stay in Mexico. He also reported that intermittent high-grade fever with night sweats began shortly after his return.

The patient denied chest pain, visual disturbance, diarrhea, constipation, dysuria, hematuria, hematochezia, or melena.

At presentation, he was noted to have a high-grade fever of 103.2 °F, while the remaining vital signs were stable. Physical examination was significant for RUQ tenderness to palpation with a positive Murphy sign; the remainder of the examination was unremarkable.

The patient was empirically started on IV ceftriaxone 2 g every 24 hours and vancomycin infusion, along with fluid resuscitation. Due to his recent travel history, consumption of undercooked pork, and recent tattoo, a broad differential diagnosis was considered, including cysticercosis, tick-borne illness, mycobacterial infections, parasitic etiologies, syphilis, malignancy, and human immunodeficiency virus. Laboratory workup during hospitalization is summarized in Table 1. 

**Table 1:** Laboratory investigations. PCR: polymerase chain reaction, TB: tuberculosis, HIV: human immunodeficiency virus, DNA: deoxyribonucleic acid, HSV: herpes simplex virus.

Test Name	Result	Reference Range
Complete blood count (CBC)		
White blood cell count	10.9 k/µL	4.2-9.1 k/µL
Red blood cell count	4.80 million/µL	4.63-6.08 million/µL
Hemoglobin	12.8 g/dL	13.7-17.5 g/dL
Hematocrit	40.2%	40.1-51.0%
Mean corpuscular volume	83.8 fL	79.0-92.2 fL
Mean corpuscular hemoglobin	26.7 pg	25.7-32.2 pg
Mean corpuscular hemoglobin concentration	31.8 g/dL	32.3-36.5 g/dL
Red cell distribution width	14.8%	11.6-14.4%
Platelet count	223 k/µL	163-337 k/µL
Mean platelet volume	10.2 fL	9.4-12.4 fL
Neutrophils	86%	34-71%
Lymphocytes	5%	19-53%
Monocytes	8%	5-13%
Eosinophils	0%	1-7%
Basophils	0%	0-1%
Immature granulocytes	1%	0-1%
Absolute neutrophil count	9.45 k/µL	1.05-6.10 k/µL
Absolute lymphocyte count	0.52 k/µL	1.10-3.70 k/µL
Absolute monocyte count	0.89 k/µL	0.20-0.80 k/µL
Absolute eosinophil count	0.00 k/µL	0.00-0.50 k/µL
Absolute basophil count	0.02 k/µL	0.00-0.08 k/µL
Absolute immature granulocyte count	0.05 k/µL	0.00-0.31 k/µL
Comprehensive metabolic panel (CMP)		
Sodium	133 mmol/L	136-145 mmol/L
Potassium	3.8 mmol/L	3.5-5.1 mmol/L
Chloride	99 mmol/L	98-107 mmol/L
Carbon dioxide	21 mmol/L	22-29 mmol/L
Blood urea nitrogen	16 mg/dL	6-20 mg/dL
Creatinine	1.00 mg/dL	0.67-1.17 mg/dL
Glucose	108 mg/dL	74-99 mg/dL
Calcium	8.8 mg/dL	8.6-10.0 mg/dL
Total protein	6.8 g/dL	6.4-8.3 g/dL
Albumin	3.7 g/dL	4.0-4.9 g/dL
Total bilirubin	0.5 mg/dL	0.0-1.2 mg/dL
Alkaline phosphatase	52 U/L	40-129 U/L
Aspartate aminotransferase	13 U/L	0-40 U/L
Alanine aminotransferase	13 U/L	≤41 U/L
Estimated glomerular filtration rate	>60 mL/min/1.73 m²	≥60 mL/min/1.73 m²
Other laboratory evaluations		
Magnesium	1.5 mg/dL	1.6-2.6 mg/dL
Lipase	27 U/L	13-60 U/L
Lactic acid	1.7 mmol/L	<2.0 mmol/L
Troponin	<6 ng/dL	≤15 ng/dL
Erythrocyte sedimentation rate	57 mm/h	0-14 mm/h
C-reactive protein	131.8 mg/L	≤5.0 mg/L
Microbiology/infectious disease		
Respiratory pathogen PCR panel	Negative	Not detected
Blood cultures (2 sets)	No growth	No growth
HIV detection with reflex confirmation	Nonreactive	Nonreactive
QuantiFERON-TB Gold Plus	Negative	Negative
Tickborne PCR panel	Not detected	Not detected
Treponema pallidum antibodies	Negative	Negative
Acute hepatitis panel	Negative	Negative
Epstein-Barr virus DNA by PCR	318 copies/mL	Not detected
Blood parasite smear (malaria)	Negative	Negative
Stool ova and parasite screen	Negative	Negative
Histoplasma urine antigen	<0.2 ng/mL	<0.2 ng/mL
Histoplasma antibody	<1:8	<1:8
Beta-D-glucan	Negative	Negative
Aspergillus antigen	Not detected	Not detected
Cryptococcal antigen (blood)	Negative	Negative
Blastomyces urine antigen	Negative	Negative
Herpes simplex virus PCR (HSV-1, HSV-2)	Negative	Negative

Autoimmune panel results (double-stranded deoxyribonucleic acid, anti-Smith, ribonucleoprotein, Sjögren, scleroderma-70, anti-centromere, ribosomal P protein, rheumatoid factor, and cyclic citrullinated peptide) were negative. Cytoplasmic and perinuclear anti-neutrophil cytoplasmic antibody levels were also normal.

Contrast-enhanced computed tomography (CT) of the abdomen revealed areas of biliary dilatation throughout the liver consistent with Caroli disease (Figure 1). 

**Figure 1:**
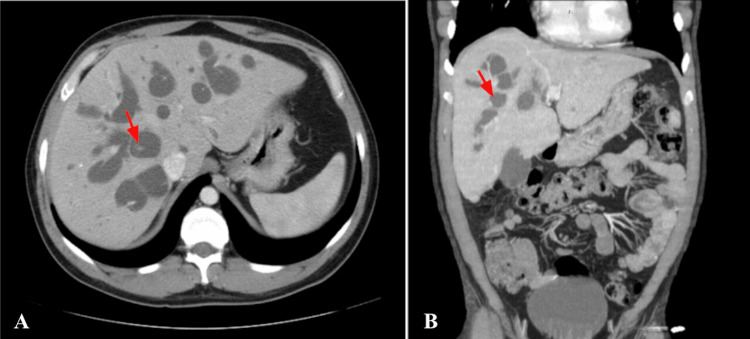
Contrast-enhanced computed tomography of the abdomen demonstrating Caroli disease. (A) Axial CT image in the portal venous phase showing multiple low-density, rounded cystic lesions scattered throughout the hepatic parenchyma, representing saccular dilatations of the intrahepatic bile ducts. Foci of contrast enhancement are seen within several of the cystic dilatations (arrow), suggestive of the "central dot sign," a finding considered highly specific for Caroli disease. (B) Coronal reformatted image confirming the diffuse intrahepatic cystic ductal dilatations with predominant involvement of the right hepatic lobe (arrow). The gallbladder is nondistended and unremarkable.

Abdominal ultrasonography revealed multiple hepatic cysts with cystic dilatation of the intrahepatic ducts, without gallbladder distention (Figure 2). 

**Figure 2:**
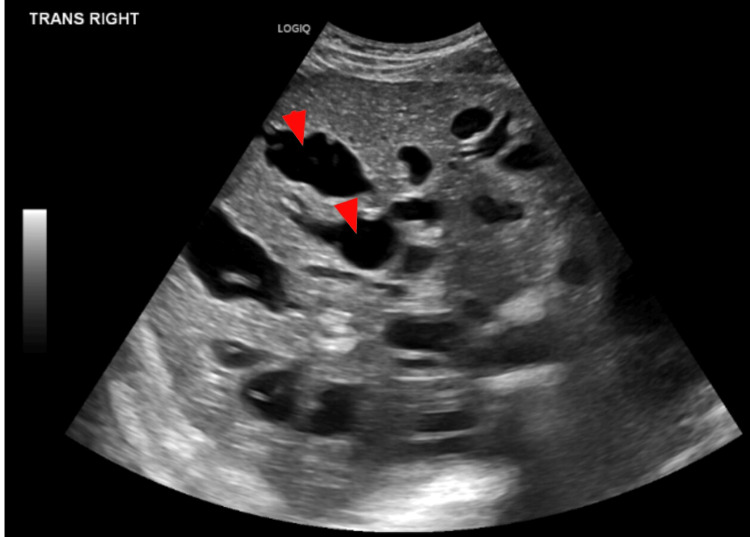
Transverse right upper quadrant ultrasonography demonstrating Caroli disease. Gray-scale ultrasonography in the transverse plane of the right hepatic lobe reveals multiple anechoic, well-defined cystic structures (arrowheads) representing saccular dilatations of the intrahepatic bile ducts, without internal echoes or septations.

A hepatobiliary iminodiacetic acid (HIDA) scan was negative for acute cholecystitis. A magnetic resonance imaging (MRI) scan of the abdomen confirmed multiple non-enhancing dilated hepatic ducts and cysts (Figure 3). 

**Figure 3:**
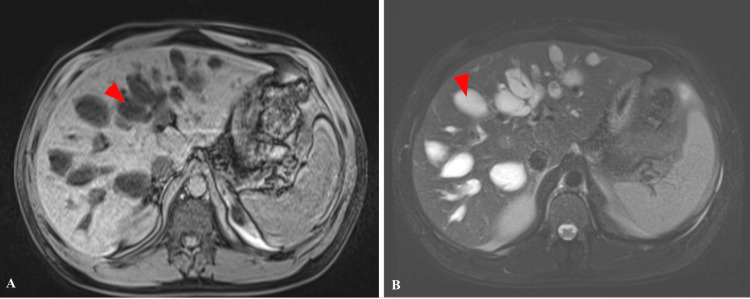
Magnetic resonance imaging of the abdomen demonstrating Caroli disease. (A) Axial T1-weighted post-contrast image showing multiple hypointense, saccular dilatations of the intrahepatic bile ducts (arrowhead) without enhancing walls or peripheral rim enhancement. (B) Axial T2-weighted fat-saturated image at the same level demonstrating the corresponding markedly hyperintense cystic ductal dilatations (arrowhead), consistent with fluid-filled, non-communicating saccular ectasia of the intrahepatic biliary tree, the characteristic MRI appearance of Caroli disease.

After initiating broad-spectrum antibiotics with vancomycin and ceftriaxone, he had rapid defervescence within 24 hours. The patient's clinical presentation was attributed to presumed recurrent cholangitis and cystic duct infection related to biliary stasis within ectatic intrahepatic ducts despite negative blood cultures.

A diagnostic breakthrough occurred when the family disclosed that the patient had been treated multiple times in the past for similar high-grade fevers at other hospitals. However, no formal diagnosis had ever been made because of the inability to follow up secondary to financial constraints.

Although he had a small subcentimeter renal cyst on each side, imaging findings and family history were negative for polycystic kidney disease.

A liver biopsy was also performed because of the patient's multiple hospitalizations for high-grade fever. Histopathological examination revealed hepatic fibrosis and disruption of the hepatic architecture by fibrous bands containing cystically dilated bile ducts, chronic inflammatory infiltrate predominantly composed of lymphocytes, trichrome staining accentuating fibrous connective tissue, and absence of stainable iron on iron staining, all consistent with the hallmark features of congenital hepatic fibrosis (portal-to-portal bridging fibrosis, bile duct proliferation, portal tract expansion, and congenital hepatic fibrosis without evidence of cirrhosis, malignancy, granulomatous inflammation, or significant steatosis), thus confirming the diagnosis of CS and differentiating it from Caroli disease. Histology images were not available due to system factors.

With his clinical improvement on broad-spectrum antibiotics, he was deemed stable for discharge on oral antibiotics for possible infected cystic ducts. Due to the final diagnosis of CS and the high risk of liver cirrhosis and potential need for liver transplantation, the patient was referred for outpatient follow-up with gastroenterology. He was also referred for outpatient follow-up with infectious disease.

## Discussion

CS is a rare fibropolycystic liver disorder resulting from ductal plate malformation and characterized by the coexistence of non-obstructive intrahepatic bile duct dilatation and congenital hepatic fibrosis. It is part of a broader spectrum of ciliopathies involving abnormalities in cholangiocyte cilia and biliary morphogenesis. The estimated incidence is approximately one per million, with most patients presenting in early adulthood, although diagnosis may be delayed into later decades because of its heterogeneous and often subtle clinical manifestations [1,2].

A key feature of CS is its highly variable clinical presentation, which frequently leads to diagnostic delay. Patients may present with either complications of bile stasis, such as recurrent cholangitis and hepatolithiasis, or manifestations of portal hypertension due to underlying hepatic fibrosis. Notably, there are no pathognomonic clinical or laboratory findings, and symptoms may be intermittent or nonspecific. A retrospective analysis demonstrated that the absence of characteristic features often results in prolonged diagnostic timelines, particularly in patients with recurrent unexplained febrile episodes. In this context, our patient's history of repeated hospitalizations with high-grade fevers prior to diagnosis closely mirrors patterns described in the literature [3].

The pathophysiology of recurrent fever in CS is primarily driven by bile stasis within ectatic intrahepatic ducts, which predisposes to bacterial colonization and recurrent cholangitis. This process may occur even in the absence of overt biliary obstruction or abscess formation, leading to intermittent or localized infections that may not always be detected on imaging or microbiological studies. Recent literature emphasizes that recurrent cholangitis in fibropolycystic liver diseases can be clinically significant despite negative cultures, particularly when infection remains confined to dilated ducts [4]. This mechanism explains the clinical course observed in our patient, including high inflammatory markers, negative blood cultures, and rapid defervescence following empiric antibiotic therapy.

An important distinguishing feature in this case is the absence of significant cholestatic liver enzyme abnormalities despite clear structural biliary disease. Although elevated alkaline phosphatase and bilirubin are commonly reported in cholangitis, several studies have demonstrated that laboratory findings in CS may be normal or only minimally abnormal, particularly in early or intermittent disease states [5]. This discordance between biochemical and structural pathology underscores the importance of maintaining a high index of suspicion and proceeding to imaging in patients with recurrent RUQ pain and systemic inflammation.

Imaging plays a central role in the diagnosis of cystic liver diseases, including CS, infectious cysts, and malignant cysts, with ultrasonography, computed tomography, and magnetic resonance imaging demonstrating characteristic saccular or fusiform dilatation of the intrahepatic bile ducts. However, imaging alone cannot reliably distinguish Caroli disease from Caroli syndrome. Histopathological confirmation of congenital hepatic fibrosis remains essential for definitive diagnosis, particularly in atypical presentations [6]. In our case, liver biopsy demonstrating fibrous portal expansion and cystically dilated bile ducts confirmed the diagnosis, consistent with current diagnostic standards.

Another important aspect highlighted by this case is the risk of long-term complications associated with CS. Chronic bile stasis and recurrent inflammation predispose patients to progressive hepatic fibrosis, portal hypertension, and a significantly increased risk of cholangiocarcinoma. A recent systematic review reported an overall malignancy incidence of approximately 6.6%, with some studies suggesting rates as high as 37% in selected populations [7]. These findings emphasize the importance of early diagnosis and longitudinal surveillance, even in patients with initially mild or intermittent symptoms.

Furthermore, CS is increasingly recognized as part of a broader spectrum of fibropolycystic diseases associated with genetic mutations affecting ciliary function, particularly involving the PKHD1 gene. These disorders frequently demonstrate multiorgan involvement, including renal cystic disease, although this association was not prominent in our patient [8]. The absence of significant renal involvement in this case highlights the phenotypic variability of CS and reinforces that its diagnosis should not be excluded in the absence of classic systemic associations.

In comparison with previously reported series, this case is notable for three key features: presentation as fever of unknown origin with an extensive negative infectious workup, absence of biochemical cholestasis despite significant biliary abnormalities, and delayed diagnosis despite recurrent episodes. These findings align with emerging literature suggesting that CS may frequently masquerade as infectious or inflammatory conditions, leading to repeated hospitalizations and extensive but unrevealing investigations [9]. Recognition of this pattern is critical for timely diagnosis and prevention of complications.

## Conclusions

CS can present with recurrent fevers, abdominal pain, and normal liver enzyme levels, a deceptive combination that poses diagnostic challenges. A high index of suspicion for CS is recommended in patients with recurrent febrile episodes without a clear cause and RUQ pain, even in the absence of transaminase elevation. Magnetic resonance cholangiopancreatography (MRCP), complemented by contrast-enhanced CT, can identify the characteristic intrahepatic biliary ectasia and biliary communication patterns associated with CS. However, liver biopsy is necessary to confirm congenital hepatic fibrosis, a hallmark of CS, particularly in cases of diagnostic uncertainty. It also guides long-term surveillance for cholangiocarcinoma and decisions regarding liver transplantation.
